# Comparison of calculated and experimental isotope edited FTIR difference spectra for purple bacterial photosynthetic reaction centers with different quinones incorporated into the Q_A_ binding site

**DOI:** 10.3389/fpls.2013.00328

**Published:** 2013-08-30

**Authors:** Nan Zhao, Hari P. Lamichhane, Gary Hastings

**Affiliations:** Department of Physics and Astronomy, Georgia State UniversityAtlanta, GA, USA

**Keywords:** ONIOM, FTIR, QA, quinone, reaction center, density functional theory (DFT) calculations

## Abstract

Previously we have shown that ONIOM type (QM/MM) calculations can be used to simulate isotope edited FTIR difference spectra for neutral ubiquinone in the Q_A_ binding site in *Rhodobacter sphaeroides* photosynthetic reaction centers. Here we considerably extend upon this previous work by calculating isotope edited FTIR difference spectra for reaction centers with a variety of unlabeled and ^18^O labeled foreign quinones incorporated into the Q_A_ binding site. Isotope edited spectra were calculated for reaction centers with 2,3-dimethoxy-5,6-dimethyl-1,4-benzoquinone (MQ_0_), 2,3,5,6-tetramethyl-1, 4-benzoquinone (duroquinone, DQ), and 2,3-dimethyl-l,4-naphthoquinone (DMNQ) incorporated, and compared to corresponding experimental spectra. The calculated and experimental spectra agree well, further demonstrating the utility and applicability of our ONIOM approach for calculating the vibrational properties of pigments in protein binding sites. The normal modes that contribute to the bands in the calculated spectra, their composition, frequency, and intensity, and how these quantities are modified upon ^18^O labeling, are presented. This computed information leads to a new and more detailed understanding/interpretation of the experimental FTIR difference spectra. Hydrogen bonding to the carbonyl groups of the incorporated quinones is shown to be relatively weak. It is also shown that there is some asymmetry in hydrogen bonding, accounting for 10–13 cm^−1^ separation in the frequencies of the carbonyl vibrational modes of the incorporated quinones. The extent of asymmetry in H-bonding could only be established by considering the spectra for various types of quinones incorporated into the Q_A_ binding site. The quinones listed above are “tail-less.” Spectra were also calculated for reaction centers with corresponding “tail” containing quinones incorporated, and it is found that replacement of the quinone methyl group by a phytyl or prenyl chain does not alter ONIOM calculated spectra.

## Introduction

Quinones play an important role in biological proton and electron transfer processes that occur in both respiration and photosynthesis (Trumpower, 1982). In type II photosynthetic reaction centers two quinone molecules act as terminal electron acceptors (Ke, 2001a,b). The two quinones are often termed Q_A_ and Q_B_. In this manuscript we will refer to the quinone binding site as Q_A_ and Q_B_, however. The quinones that occupy the Q_A_ and Q_B_ binding sites have very different functions. The Q_A_ quinone is an intermediary cofactor involved in transferring electrons from (bacterio) pheophytin to Q_B_, while the Q_B_ quinone couples proton and electron transfer processes (Ke, 2001b,c,d).

In this manuscript we focus on the Q_A_ binding site. The quinone occupying the Q_A_ binding site is species dependent. In *Rhodobacter (Rb.) sphaeroides* purple bacterial reaction centers (PBRCs) a ubiquinone (UQ) molecule occupies the Q_A_ binding site. In PBRCs from *Blastochloris viridis* (Shopes and Wraight, 1985) and *Chloroflexus aurantiacus* (Hale et al., 1983) a menaquinone occupies the Q_A_ binding site. In photosystem II reaction centers from oxygen evolving organisms, a plastoquinone (PQ) molecule occupies the Q_A_ binding site. In photosystem I the secondary electron acceptor, termed A_1_, is a vitamin K_1_ (VK) molecule (also called phylloquinone). Menaquinone and VK are both naphthoquinone (NQ) moieties that differ only in the degree of saturation of the tail at C_6_.

**Figure 1:**
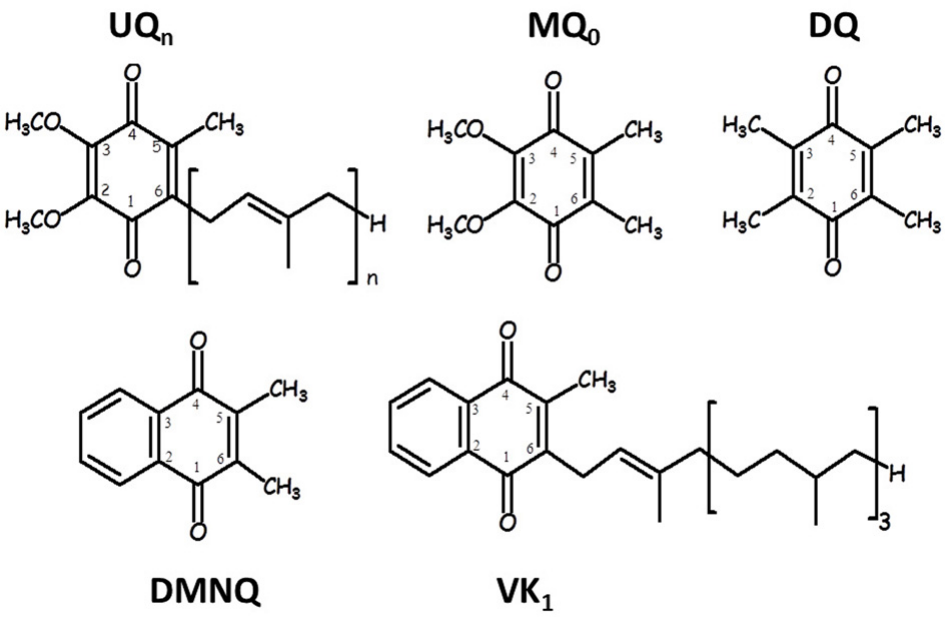
**Structure and numbering of ubiquinone (2,3-dimethoxy, 5-methyl,6-prenyl benzoquinone) (UQ_*n*_), 2,3-dimethoxy, 5,6-methyl benzoquinone (MQ_0_), 2,3,5,6-methyl benzoquinone (duroquinone, DQ), 2,3-dimethyl, 1,4-naphthoquinone (DMNQ) and 2-methyl, 3-phytyl 1,4-naphthoquinone (VK)**. The numbering scheme employed here for the naphthoquinone structures is nonstandard, and was chosen to facilitate comparison between naphthoquinone and ubiquinone structures.

Figure 1 shows the structure, numbering and abbreviations we will use for the various quinones discussed in this manuscript. MQ_0_ and DMNQ are UQ and VK analogues, respectively, in which the hydrocarbon tail has been replaced with a methyl group. DQ is a PQ analogue in which the hydrocarbon chain at C_6_ is replaced with a methyl group.

It has been suggested, at least for the UQ that occupies the Q_A_ site in PBRCs, that the role of the hydrocarbon chain at C_6_ is to anchor and orient the quinone head-group in a specific way (Warncke et al., 1994). Data is available that may argue against this proposal, however, (McComb et al., 1990; Srinivasan and Golbeck, 2009; Wraight and Gunner, 2009).

Comparison of the properties of PBRCs with MQ_0_ and UQ_*n*_, or VK and DMNQ, incorporated into the Q_A_ binding site will allow one to assess how or if the hydrocarbon chain at C_6_ modifies the quinones functional properties. Similarly, comparison of the properties of PBRCs with MQ_0_ and DQ incorporated into the Q_A_ binding site will allow one to assess how or if the methoxy groups at C_2_ and C_3_ modifies the quinones functional properties. Calculated spectra for *Rb. sphaeroides* PBRCs with VK in the Q_A_ binding site can be compared to experimental spectra. These calculated spectra may also serve as a useful model for *B. viridis* PBRCs that naturally have VK incorporated into the Q_A_ binding site.

Fourier transform infrared (FTIR) difference spectroscopy (DS) is a sensitive molecular-level probe of pigment-protein interactions, and it is widely used to study both the neutral and reduced states of quinones in PBRCs (Breton and Nabedryk, 1996) and in photosystem II (Noguchi and Berthomieu, 2005). In this manuscript we focus on Q^−^_A_/Q_A_ FTIR DS. Many molecular species contribute to Q^−^_A_/Q_A_ FTIR DS, and in the past it has been difficult to identify which bands are associated specifically with UQ in the Q_A_ site. However, fully functional quinones can be incorporated into Q_A_ depleted PBRCs, and by collecting Q^−^_A_/Q_A_ FTIR DS using PBRCs with unlabeled and isotopically labeled quinones incorporated, so called isotope edited FTIR difference spectra can be constructed, and from these spectra it has proven possible to separate contributions of the quinones from those of the protein in Q^−^_A_/Q_A_ FTIR DS (Breton and Nabedryk, 1996). Previously, a variety of unlabeled and ^18^O labeled quinones have been incorporated into the Q_A_ binding site in PBRC's, and ^18^O isotope edited FTIR DS have been obtained (Breton et al., 1994a). The goal in this manuscript is the simulation of these experimental ^18^O isotope edited FTIR DS associated with the neutral state of the quinone in the Q_A_ binding site. Calculated IR spectra associated with the quinone anion state are considerably more complicated (Lamichhane and Hastings, 2013) and are currently being undertaken.

Although experimental Q^−^_A_/Q_A_ FTIR DS have been obtained using PBRCs with various unlabeled and isotope labeled quinones incorporated, virtually no computational work aimed at modeling the experimental FTIR DS have been undertaken. Calculations aimed at modeling the vibrational properties of quinones in the Q_A_ binding site must account for the protein environment. Quantum mechanical (QM) calculations of such a large molecular system (pigment plus protein environment) are unfeasible. To circumnavigate this problem methods have been developed that allow one to separate the molecular system into distinct layers that can be treated at different levels of theory. In one layer the chemical properties of the principle species of interest (the pigment) can be calculated using “high-level” QM methods. The surrounding protein environment is included in the calculation but it is treated using computationally less expensive molecular mechanics (MM) methods.

Recently, we have undertaken QM:MM calculations for UQ in the Q_A_ binding site using the ONIOM method (Vreven et al., 2006). ONIOM is an acronym for: our Own N-layered Integrated molecular Orbital + Molecular mechanics package. In these calculations we showed that we could simulate experimental isotope edited FTIR difference spectra obtained using PBRCs with neutral UQ in the Q_A_ binding site. Here we extend upon these previous studies by attempting to simulate experimental isotope edited FTIR difference spectra obtained using PBRCs with symmetric tail-less and corresponding tail containing quinones incorporated into the Q_A_ binding site. We show that the calculated spectra agree well with the experimental spectra, further supporting the notion that the ONIOM method is a useful approach for understanding complex FTIR difference spectra associated with pigments in protein binding sites. We are also able to assess to what extent the quinone hydrocarbon chain may influence the calculated spectra.

## Materials and methods

### Structural model used in calculations

The molecular model used in ONIOM calculations was generated from the crystal structure of *Rb*. *sphaeroides* PBRCs at 2.2 Å resolution (Stowell et al., 1997) (PDB file 1AIJ). From the PDB file all atoms within 10 Å of either carbonyl oxygen atom of UQ were selected. This subset of atoms formed the basis of the Q_A_ binding site structural model. Hydrogen atoms (not included in the PDB file) were added to the model using the software Gaussview4, resulting in a final structural model consisting of 1024 atoms. Following the addition of hydrogen atoms the structural model was optimized (energy minimized) using ONIOM methods with all atoms associated with the protein backbone, and the non-heme iron atom, being held fixed. All atoms of the amino acid side chains and the incorporated quinone are unconstrained. For calculations of UQ/VK the hydrocarbon tail was modeled as a single prenyl/phytyl unit, respectively. Inclusion of further prenyl/phytyl units did not alter the calculated spectra (not shown).

**Figure 2:**
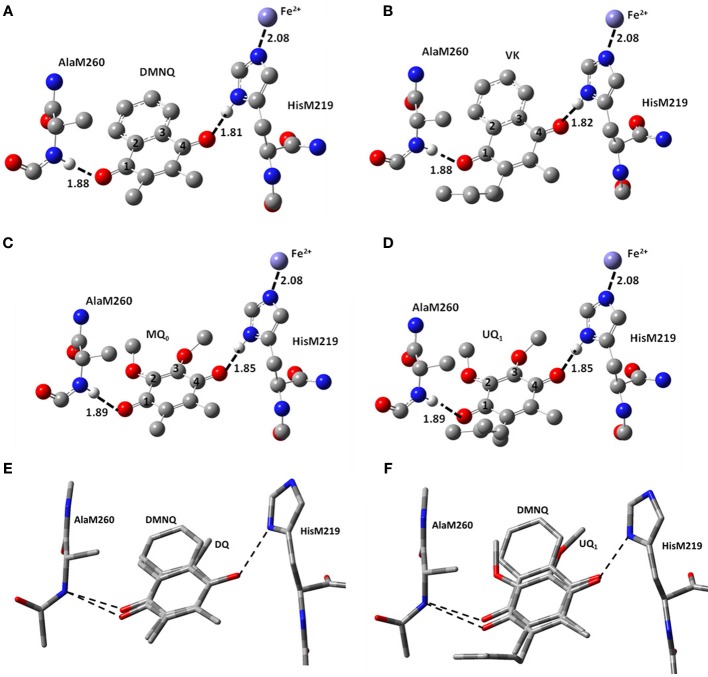
**Ball and stick representation of the calculated structure of (A) DMNQ, (B) VK, (C) MQ_0_ and (D) UQ_1_ in the Q_A_ binding site in PBRCs from *Rb. sphaeroides***. Structure shown is after geometry optimization using ONIOM methods. Possible H-bonds are shown (dotted). Hydrogen atoms, except the ones involved in H-bonding, have been omitted. Distances quoted are in Å. **(E,F)** Overlap of structures obtained from ONIOM calculations for **(E)** DMNQ and DQ and **(F)** DMNQ and UQ_1_. Overlap is based on using the (fixed) backbone atoms of HisM219 and AlaM260. The amino acid side chains are virtually unaltered in the structures shown.

The structural models for the different incorporated quinones are initially set up by simply replacing the molecular substituents of the originally incorporated UQ species. So, the C=O groups of the different quinones incorporated will initially have the same orientation and position to that found for UQ in the Q_A_ binding site. DMNQ and VK structures were constructed starting from the UQ structure, by replacing the methoxy groups with the NQ aromatic ring, without alteration of the quinone ring (Figure 2F). This orientation of the NQ ring (of DMNQ and VK) was chosen as previous docking calculations have suggested it is the most favorable (Hucke et al., 2002).

### Calculations

All calculations were undertaken using Gaussian 03 (Frisch et al., 2004) software. For calculation of UQ molecules in the gas phase, and for the QM part of ONIOM calculations, molecular geometry optimizations and harmonic vibrational frequency calculations were undertaken using hybrid DFT methods, employing the B3LYP functional and the 6–31+G(d) basis set. This choice of functional and basis set are appropriate for calculation of the vibrational properties of quinones (Wheeler, 2001; Bandaranayake et al., 2006). The MM part of the ONIOM calculation is undertaken using the AMBER force field (Case et al., 2005). Following ONIOM geometry optimization of the structural model, the optimized quinone molecule from the model is considered separately for vibrational frequency calculations.

### Normal mode assessment

Assignment of calculated vibrational frequencies to molecular groups is based on a consideration of the calculated atomic displacements (in Cartesian coordinates) associated with the normal modes. These atomic displacements can be animated using software (GaussView4), and the molecular groups that most prominently contribute to the normal modes can be assessed visually [see Figure 2 in Bandaranayake et al. (2006)]. In addition, potential energy distributions (PEDs) of the normal modes are calculated using the freeware GAR2PED (Martin and Van Alsenoy, 1995). For the various quinones in the Q_A_ binding site we calculate both vibrational mode frequencies and intensities. With both the frequency and intensity information “stick spectra” can be constructed. These stick spectra are representative of IR absorption spectra, as described previously (Bandaranayake et al., 2006; Parameswaran et al., 2008; Lamichhane et al., 2010). The calculated stick spectra are convolved with a Gaussian function with half-width of 4 cm^−1^ to produce more realistic looking IR absorption spectra (Bandaranayake et al., 2006; Parameswaran et al., 2008; Lamichhane et al., 2010).

## Results

### UQ structure and numbering

Figure 1 shows the structure and numbering scheme used here for UQ_*n*_, MQ_0_, DQ, DMNQ, and VK. NQ's generally have a different numbering scheme. We have applied the UQ numbering scheme to DMNQ and VK for the sake of easy comparison. Figure 2 shows a picture of **(A)** DMNQ, **(B)** VK, **(C)** MQ_0_, and **(D)** UQ_1_ in the Q_A_ binding site along with the two H-bonding amino acids. The structures shown are after geometry optimization using ONIOM methods. Possible hydrogen bonds (or ligand to the non-heme iron atom) are indicated by dotted lines.

To gain a better sense of the relative orientation of the different quinones in the Q_A_ binding site Figure 2 also shows the **(E)** DMNQ/DQ and **(F)** DMNQ/UQ structures from two ONIOM calculations overlapped. These overlapped structures are created by considering the (fixed) atoms of the protein backbone. Figures 2E,F indicates that the side chains of HisM219 and AlaM260 are unaltered when a different quinone is incorporated into the binding site.

Table 1 lists several bond lengths and bond angles derived from our ONIOM calculated optimized geometries of the various quinones in the Q_A_ binding site. For comparison, Table 1 also lists corresponding bond lengths and angles derived from our DFT calculated optimized geometries of the various quinones in the gas phase. Table 1 also list results obtained from previous QM/MM calculations (Sinnecker et al., 2006), and data taken from the 1AIJ crystal structure (Stowell et al., 1997).

**Table 1:** **Comparison of bond lengths and angles derived from the ONIOM calculated (O) and gas phase calculated (GP) optimized geometry of neutral UQ_1_, MQ_0_, DQ, VK, and DMNQ**.

	**x-ray**	**UQ1**		**MQo**		**DQ**		**VK_1_**		**DMNQ**		**Ref**
		**O**	**GP**	**O**	**GP**	**O**	**GP**	**O**	**GP**	**O**	**GP**	**UQ1**
C_1_=O	1.234	1.227	1.223	1.227	1.223	1.232	1.230	1.233	1.229	1.232	1.229	1.235
C_4_=O	1.232	1.238	1.231	1.238	1.231	1.236	1.231	1.236	1.229	1.236	1.229	1.237
C_2_=C_3_	1.404	1.366	1.364	1.367	1.364	1.355	1.355	1.405	1.405	1.406	1.405	
C_5_=C_6_	1.419	1.354	1.354	1.354	1.354	1.357	1.355	1.363	1.361	1.362	1.360	
**AlaM260 H-BOND**												
N–O	2.837	2.837		2.846		2.895		2.856		2.878		
N-H	1.014	1.014		1.014		1.013		1.013		1.013		1.014
N-H–O	1.912	1.894		1.886		1.907		1.883		1.883		1.94 ± 0.11
∠ N-H-O	150.2	153.3		156.7		164.4		159.9		166.7		147 ± 7
∠ C-O–N	131.5	127.0		125.9		127.4		125.7		124.1		
∠ C-O-H	133.4	128.7		128.3		132.6		128.0		128.1		
**HisM219 H-BOND**												
N–O	2.788	2.868		2.865		2.829		2.832		2.831		
N-H	1.020	1.020		1.020		1.019		1.020		1.020		1.017
N-H–O	1.788	1.849		1.845		1.810		1.815		1.812		1.79 ± 0.14
∠-N-H–O	166.2	175.8		177.7		178.8		174.3		178.2		170 ± 6
∠ C-O–N	138.4	140.1		139.7		137.4		138.4		137.8		
∠ C-O–H	140.6	139.4		139.2		137.4		137.5		137.3		
C_4_=O − Fe	6.832	6.744		6.715		6.627		6.692		6.637		
∠ Tail	113.0	114.9	111.7					114.7	111.6			
C_2_-dihedral	−57.1	−25.3	−8.9	−26.5	−10.1							
C_3_-dihedral	109.5	150.5	123.6	150.6	123.1							

Bond lengths and angles from the 1AIJ crystal structure (Stowell et al., 1997) are also listed. Distances are in Å and angles are in degrees. The C_2_ and C_3_ methoxy group dihedral angles are defined as the C_3_-C_2_-O-CH_3_ and C_2_-C_3_-O-CH_3_ dihedral angles from Sinnecker et al. (2006).

Table 1 list the three distances associated with the peptide or imidazole H-bond to the carbonyl oxygen atoms of the quinone (N-H, NH–O and N–O distances). These distances completely determine the N-H–O H-bond geometry. Associated angles are also listed in Table 1.

For UQ_10_ in the Q_A_ site the 1AIJ crystal structure (Stowell et al., 1997) indicates that the C_1_=O bond is marginally longer than the C_4_=O bond. (1.234 vs. 1.232 Å). In contrast, from ONIOM calculations of all the quinones listed in Table 1 the C_1_=O bond is shorter than the C_4_=O bond.

In gas phase calculations the C_1_=O and C_4_=O bond lengths are shorter than that found in ONIOM calculations (except the C_1_=O of DQ). This lengthening of the C=O bonds of the quinones in the Q_A_ binding site is related to hydrogen bonding and other electrostatic interactions of the pigment with the protein environment.

For UQ_1_ and MQ_0_ the C_1_=O bond is shorter than the C_4_=O bond in both ONIOM and gas phase calculations. In gas phase calculations this difference in C_1_=O and C_4_=O bond lengths of UQ_1_ and MQ_0_ must relate to the differing orientations of the C_2_ and C_3_ methoxy groups. The ONIOM calculated C_2_ and C_3_ methoxy group dihedral angles for UQ_1_ are −25.3 and 150.5° (Table 1). Similar angles are calculated for MQ_0_. The calculated dihedral angles for UQ_1_ in the gas phase, and the observed angles for UQ_10_ in the Q_A_ binding site (from the crystal structure) are within 32° of that calculated for UQ_1_ using ONIOM methods.

For UQ_10_ in the Q_A_ site the crystal structure indicates that the C_2_=C_3_ bond is shorter than the C_5_=C_6_ bond. (1.404 vs. 1.419 Å). In contrast, for all quinones except DQ, in both ONIOM and gas phase calculations the C_2_=C_3_ bond is longer than the C_5_=C_6_ bond. For VK and DMNQ the calculated C_2_=C_3_ and C_5_=C_6_ bond lengths are considerably different.

For UQ_1_ and VK the hydrocarbon chain attached at C_6_ makes a distinct “kink” after the first carbon atom (Figures 2B,D). The C-C-C bond angle is 112–115° for both quinones in both the ONIOM and gas phase calculations. The calculated angles are virtually the same as that found in the crystal structure. Given these similarities (between the ONIOM and gas phase calculations for both UQ_1_ and VK, as well as between calculation and experiment) the suggestion is that the protein environment does not constrain the orientation of the quinone ring relative to the C_6_ hydrocarbon chain.

**Figure 3:**
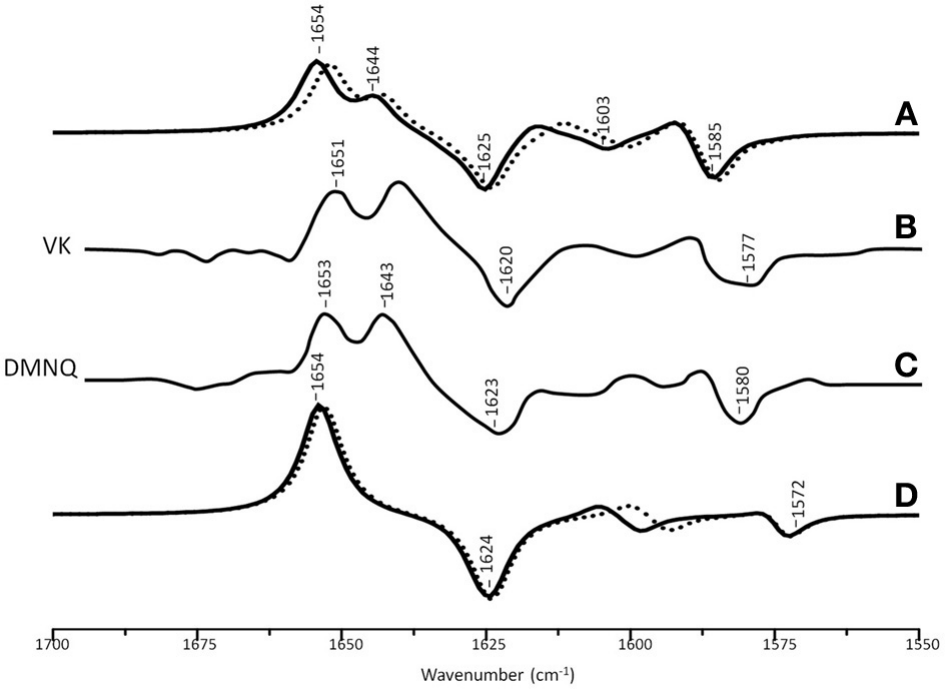
**(A)** ONIOM calculated ^18^O isotope edited DDS for neutral VK (dotted) and DMNQ (solid) in the Q_A_ binding site. Experimental spectra for VK **(B)** and DMNQ **(C)** are also shown, and were taken from Breton et al., (1994a), with permission. **(D)** DFT calculated ^18^O isotope edited DDS for neutral VK (dotted) and DMNQ (solid) are also shown. ONIOM/gas phase calculated spectra were scaled by 0.9718/0.9608, respectively.

Figure 3A shows ONIOM calculated ^18^O isotope edited difference spectra for neutral VK and DMNQ. Figure 3D shows corresponding DFT calculated spectra for VK and DMNQ in the gas phase. Experimental spectra are also shown (Figures 3B,C) for comparison. Positive/negative bands in the isotope edited spectra are due to the unlabeled/^18^O labeled quinone species, respectively. The ONIOM calculated spectra clearly better describe the experimental spectra. Calculations including the protein environment appear to be necessary in order to adequately simulate the experimental spectra.

In the DFT calculated ^18^O isotope edited spectrum for DMNQ/VK in the gas phase (Figure 3D) the antisymmetric vibration of both C=O groups gives rise to the band at ~1654 cm^−1^ (Figure 3D), which downshifts 30 cm^−1^ upon ^18^O labeling (Bandaranayake et al., 2006). The calculated isotope-edited gas phase spectrum is in excellent agreement with the experimental spectrum for DMNQ in solution (Breton et al., 1994a).

Figure 3 shows that except for a small frequency shift in some of the modes, the calculated spectra for DMNQ and VK are virtually the same. Replacement of the methyl group at C_6_ with an isoprene unit therefore, has no influence on the calculated spectra.

The normal modes (frequencies and intensities) that give rise to the various bands in the ONIOM calculated ^18^O isotope edited spectra of DMNQ and VK are listed in Table 2. The PEDs, which quantify to what extent various internal coordinates contribute to the normal modes, are also listed in Table 2. Results for DMNQ and VK are very similar. Below we will discuss calculated data obtained for DMNQ with the recognition that very similar results and conclusions also apply to VK.

**Table 2:** **Normal mode frequencies (in cm^−1^), intensities (in km/mol) and PEDs (in %) calculated using ONIOM methods for unlabeled and ^18^O labeled neutral DMNQ, VK, DQ, MQ_0_, and UQ_1_**.

**Unlabeled**				**^18^O labeled**			
	**ν**	***I***	**Potential energy distribution**	**ν**	***I***	**Δν**	**Potential energy distribution**
DMNQ	1654	235	C_1_=O (71)	1631	32	23	C_1_=O (28), C_5_=C_6_ (22), C_2_=C_3_ (10)
VK	1652	224	C_1_=O (69)	1628	29	24	C_1_=O (32), C_5_ =C_6_ (16), C_2_=C_3_ (6)
DQ	1646	254	C_1_=O (83)	1613	264	33	C_1_=O (80)
MQ_0_	1666	209	C_1_=O (81)	1631	211	35	C_1_=O (83)
UQ_1_	1663	185	C_1_=O (80)	1629	187	34	C_1_=O (82)
DMNQ	1644	108	C_4_=O (60)	1625	199	19	C_4_=O (26), −C_1_=O (21),C_5_ = C6 (8), −C_3_ = C_7_ (7)
VK	1642	107	C_4_=O (60)	1624	188	18	C_4_=O (29), −C_1_=O (15), C_5_=C_6_ (8), −C_3_ = C_7_ (7)
DQ	1632	181	C_4_=O (83)	1599	144	33	C_1_=O (81)
MQ_0_	1627	270	C_4_=O (70), −C_2_=C_3_ (8)	1587	265	40	C_4_=O (63), C_2_=C_3_ (9), −C_5_=C_6_ (5)
UQ_1_	1626	305	C_4_=O (68), −C_2_=C_3_ (9)	1586	249	40	C_4_=O (65), C2 = C3 (7),−C_5_=C_6_ (6)
DMNQ	1617	59	C_5_=C_6_ (61), −C_4_=O (8)	1604	54	13	C_5_=C_6_ (37), −C_4_=O (25), −C_1_=O (14)
VK	1611	54	C_5_=C_6_ (60), −C_4_=O (8)	1600	52	11	C_5_=C_6_ (42), −C_4_=O (20), −C_1_=O (14)
DQ	1660	1	C_2_=C_3_ (32), C_5_=C_6_ (28)	1660	0	0	C_5_=C_6_ (29), C_2_=C_3_ (33)
							
MQ_0_	1657	20	C_5_=C_6_ (52), C_2_=C_3_ (15)	1657	8	0	C_5_=C_6_ (49), C_2_=C_3_ (13)
UQ_1_	1653	26	C_5_=C_6_ (45), C_2_ = C_3_ (19)	1654	18	−8	C_5_=C_6_ (44), C_2_=C_3_ (17)
DMNQ	1591	93	C=C_arom_ (55)	1586	181	5	C=C_arom_ (43),−C_4_=O (13), C_1_=O (10)
VK	1591	96	C=C_arom_ (54),−C_4_=O (5)	1585	186	6	C=C_arom_ (40), −C_4_ = O (14), C_1_=O (11)
							
DQ	1620	55	C5 = C6 (38), −C_2_=C_3_ (33)	1620	56	0	C_5_=C_6_ (37), −C_2_=C_3_ (34)
							
MQ_0_	1601	293	C_2_=C_3_ (39), −C_5_=C_6_ (15), C_4_=O (12), −C_2_=O (7)	1609	273	−7	C_2_=C_3_ (40), −C_4_=O (15), −C_5_=C_6_ (12), −C_2_ =O (11)
UQ_1_	1601	275	C_2_=C_3_ (35), −C_5_=C_6_ (18), C_4_ =O (14), −C_2_ =O (6)	1609	308	−8	C_2_=C_3_ (37), −C_4_=O (13), −C_5_=C_6_ (15), −C_2_–O (10)

Frequency shifts upon ^18^O labeling are also listed. Negative signs in the PEDs refer to the relative phase of vibration of the internal coordinates. Only internal coordinates that contribute at least 5 % are shown. Mode frequencies were scaled by 0.9718.

In the ONIOM calculated ^18^O isotope edited spectra for DMNQ the two bands at 1654 and 1644 cm^−1^ are due to C_1_=O and C_4_=O stretching vibrations, respectively (Table 2). These bands almost certainly correspond to the at 1653 and 1643 cm^−1^ bands in the experimental spectrum (Figure 3C). So our calculations *predict* that the 1653 and 1643 cm^−1^ bands in the experimental spectrum are due to C_1_=O and C_4_=O stretching vibrations, respectively. This is in fact the first direct evidence that the 1653 and 1643 cm^−1^ bands in the experimental spectrum are due to C_1_=O and C_4_=O stretching vibrations. A direct assignment has never been made because specific ^13^C_1_ and ^13^C_4_ isotopic labeled VK or DMNQ has never been incorporated into the Q_A_ binding site in PBRCs.

In contrast to the observation of two separate C=O modes in ONIOM calculations, in gas phase calculations the two C=O modes of DMNQ are anti-symmetrically coupled (Bandaranayake et al., 2006), giving rise to a single intense band at 1654 cm^−1^ (Figure 3D).

In ONIOM calculations it is found that upon ^18^O labeling the C_1_=O and C_4_=O modes of DMNQ downshift 23 and 19 cm^−1^, to 1631 and 1625 cm^−1^, respectively (Table 2). Upon ^18^O labeling the 1625 cm^−1^ mode is more than six times more intense than the 1631 cm^−1^ mode. The 1625 cm^−1^ mode in ^18^O labeled DMNQ is due mainly to the antisymmetric coupled vibration of both C=O groups. That is, two separate C=O modes of unlabeled DMNQ couple upon ^18^O labeling. This behavior is not predicted based upon consideration of the experimental spectra, where it is “assumed” that the two C=O modes remain separate upon ^18^O labeling (Breton et al., 1994b; Breton, 1997).

A C=C mode of the quinone ring of DMNQ is found at 1617 cm^−1^. This quinonic C=C mode downshifts 13 cm^−1^, to 1604 cm^−1^, upon ^18^O labeling, with little change in intensity. The 1604 cm^−1^ mode composition in ^18^O labeled DMNQ displays considerable mixing of the C=O and C=C modes (the C=O modes account for 39% of the PED). This behavior might be expected given that the C=O and C=C modes are closer in frequency upon ^18^O labeling.

A C=C mode of the aromatic ring of DMNQ occurs at 1591 cm^−1^. This aromatic C=C mode downshifts 5 cm^−1^ to 1586 cm^−1^ upon ^18^O labeling. The 1586 cm^−1^ mode in ^18^O labeled DMNQ displays some mixing with C=O modes (23%), and the intensity of the aromatic C=C mode nearly doubles upon ^18^O labeling. The calculated C=C normal modes and their interpretation in terms of internal coordinates, as well as the calculated ^18^O induced frequency shifts are similar to that suggested on the basis of the experimental spectra (Breton et al., 1994a).

**Figure 4:**
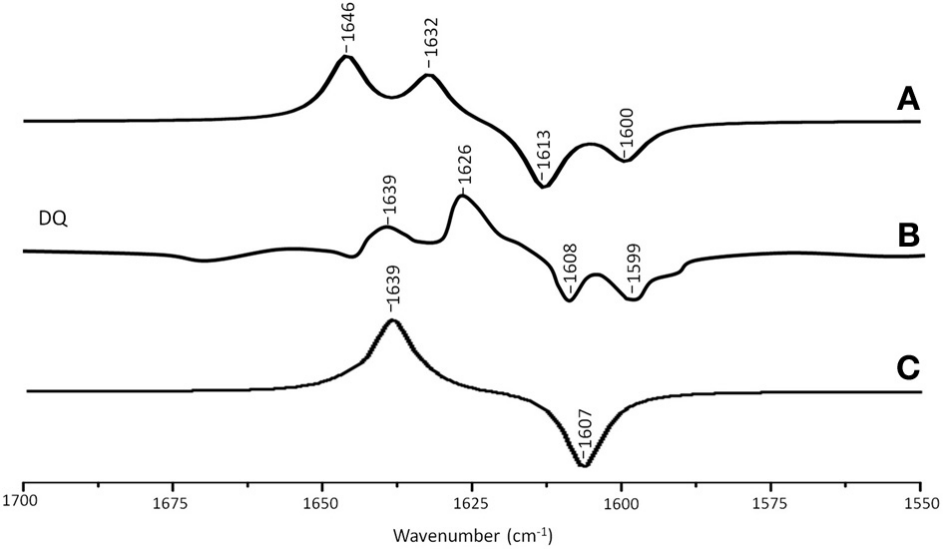
**(A)** ONIOM calculated ^18^O isotope edited DDS for neutral DQ in the Q_A_ binding site. Experimental spectra are shown in **(B)**, and were taken from Breton et al., (1994a) with permission. **(C)** DFT calculated ^18^O isotope edited DDS for neutral DQ. ONIOM and gas phase calculated spectra were scaled by 0.9718 and 0.9608, respectively.

Figure 4A shows ONIOM calculated ^18^O isotope edited IR difference spectra for neutral DQ in the Q_A_ binding site. Figure 4C shows the corresponding DFT calculated spectrum for DQ in the gas phase. The experimental spectrum is shown in Figure 4B. Again, the ONIOM calculated spectrum agrees well with the experimental spectrum while the calculated gas phase spectrum does not. The ONIOM calculated normal modes (frequencies and intensities) that give rise to the bands in the ^18^O isotope edited spectrum for DQ, as well as the PEDs, are listed in Table 2.

In the ONIOM calculated ^18^O isotope edited spectrum for DQ the two bands at 1646 and 1632 cm^−1^ are due to C_1_=O and C_4_=O stretching vibrations, respectively (Table 2). Upon ^18^O labeling the C_4_=O and C_1_=O modes both downshift 33 cm^−1^ with little change in mode intensities (Table 2). This 33 cm^−1^ downshift is large compared to that calculated for DMNQ (19–23 cm^−1^). For DQ, the ONIOM calculated mode composition is virtually unchanged upon ^18^O labeling. This is also markedly different to that calculated for DMNQ. The normal modes that give rise to the isotope edited spectra of DQ and DMNQ in the gas phase are very similar, so replacing the methyl groups of DQ with the aromatic ring of DMNQ in the Q_A_ binding site leads to an alteration in the electronic structure of the quinone ring. This modification is not obvious given the similar orientation of DQ and DMNQ in the Q_A_ binding site (Figure 2E).

In the DFT calculated ^18^O isotope edited spectrum for DQ in the gas phase, the two C=O modes are strongly coupled, and give rise to the band at 1639 cm^−1^ in Figure 4C. The calculated gas phase spectrum is in line with the experimental FTIR spectrum for DQ in solution (Breton et al., 1994a).

From ONIOM calculations for DQ, C=C modes do not couple with the C=O modes. The C_2_=C_3_ and C_5_=C_6_ groups couple to produce in phase and out of phase vibrational modes. The in phase mode has negligible IR intensity. The out of phase C=C mode is calculated to be at 1620 cm^−1^. This C=C mode is virtually unaltered in frequency, intensity, and mode composition upon ^18^O labeling (Table 2).

**Figure 5:**
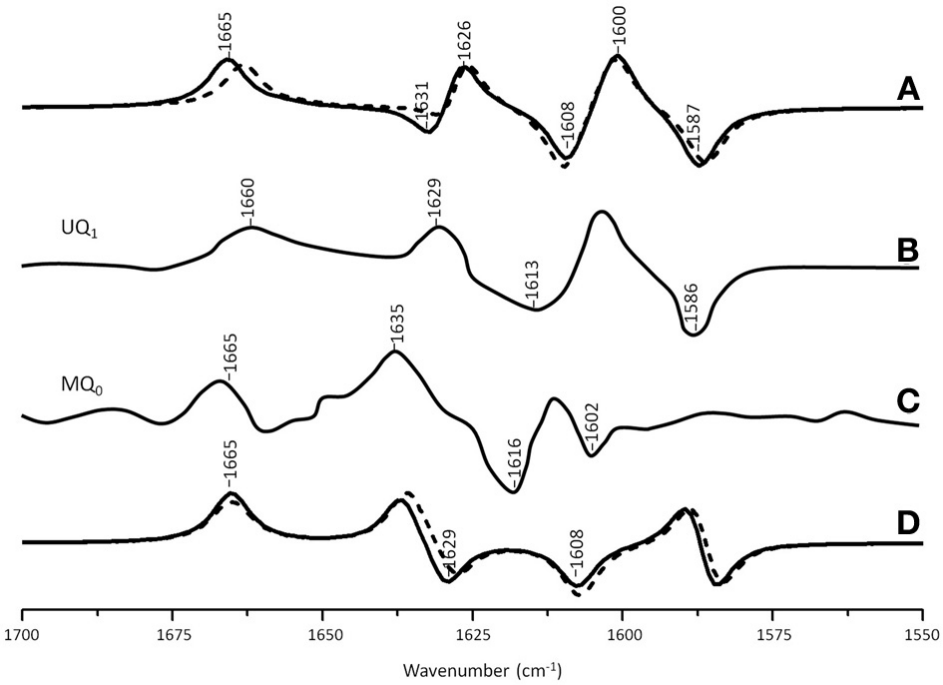
**(A)** ONIOM calculated ^18^O isotope edited DDS for neutral MQ_0_ (solid) and UQ_1_ (dotted) in the Q_A_ binding site. Experimental spectrum for **(B)** MQ_0_ and **(C)** UQ_1_ are also shown, and were taken from (Breton et al., 1994a) with permission. **(D)** DFT calculated ^18^O isotope edited DDS for neutral MQ_0_ (solid) and UQ_1_ (dotted). ONIOM and gas phase calculated spectra were scaled by 0.9718 and 0.9608, respectively.

Figure 5A shows ONIOM calculated ^18^O isotope edited IR spectra for neutral MQ_0_ (solid) and UQ_1_ (dotted) in the Q_A_ binding site. Figure 5D shows DFT calculated spectra for MQ_0_ and UQ_1_ in the gas phase. The experimental spectra for UQ_1_ and MQ_0_ are shown in Figures 5B,C, respectively. The normal modes (frequencies and intensities) that give rise to the various bands in the ONIOM calculated spectra, as well as the PEDs, are listed in Table 2. The data for UQ_1_ has been presented previously (Lamichhane and Hastings, 2011), and we show here that very similar spectra are calculated for both MQ_0_ and UQ_1_. Replacement of an isoprene unit at C_6_ with a methyl group does not greatly alter the calculated spectra.

In the ONIOM calculated ^18^O isotope edited spectra for neutral MQ_0_ the two bands at 1666 and 1627 cm^−1^ (Figure 5C) are due to C_1_=O and C_4_=O stretching vibrations, respectively (Table 2). Upon ^18^O labeling the C_1_=O and C_4_=O modes downshift 35 and 40 cm^−1^, respectively (Table 2). The mode intensities and composition are little altered by ^18^O labeling. The C=C mode of MQ_0_/UQ_1_ at 1601 cm^−1^ up-shifts 8/7 cm^−1^ upon ^18^O labeling (Table 2). An explanation for this ^18^O induced frequency upshift has been presented (Lamichhane and Hastings, 2011).

In the DFT calculated ^18^O isotope edited spectra for UQ_1_/MQ_0_ in the gas phase, the two C=O modes are well separated, and give rise to bands at ~1665 and ~1635 cm^−1^ (Figure 5D), which downshift 36 and 27 cm^−1^, respectively, upon ^18^O labeling, as described previously (Lamichhane et al., 2011).

## Discussion

Previously we have shown that ONIOM methods can be used to calculate isotope edited difference spectra for UQ_1_ in the Q_A_ binding site, and that these calculated spectra model very well the corresponding experimental spectra (Lamichhane and Hastings, 2011). Here we considerably extend these studies, and show that ONIOM calculated isotope edited spectra for different quinones in the Q_A_ binding site model very well the corresponding experimental spectra. We further show that the calculated spectra for quinones in the gas phase are totally inappropriate for modeling the vibrational properties of quinones in the Q_A_ binding site. This is also very likely to be the case for modeling the properties of any protein bound pigment.

Without normal mode vibrational frequency calculations interpretation of experimental spectra is limited, and here we clearly show that our computational methods lead to a greatly increased understanding of the normal modes that contribute to the bands in the experimental spectra.

In our ONIOM calculations for neutral UQ_1_ in the Q_A_ binding site we considered all amino acids included in the model only at the molecular mechanics level of computation. Previously, ONIOM calculations have been undertaken in order to model EPR data associated with the UQ anion in the Q_A_ binding site (Lin and O'Malley, 2008). In these calculations key amino acids, such as HisM219 and AlaM260, were considered at the higher quantum mechanical level of calculation. Taken together these results may indicate differences in the nature of H-bonding for the neutral and anion UQ species. Changes in H-bonding upon radical formation may be a mechanism for (de)stabilizing cofactors to fine-tune electron transfer processes in biological systems. Clearly it will be useful to undertake calculations similar to that presented here (modeling isotope edited FTIR difference spectra) for the UQ anion in the Q_A_ binding site, and such calculations are underway in our lab. Based on the above one could argue that the methods used here, with only the quinone treated at the QM level, will be inadequate to simulate the experimental spectra associated with quinone anions in the Q_A_ binding site. Or, for simulating the vibrational properties of quinone anions in the Q_A_ binding site it will be necessary to treat key amino acids at a quantum mechanical level. Again, calculations are underway in our lab to test this proposal.

For the neutral state of quinones occupying the Q_A_ binding site, calculations with the H-bonding amino acids treated at the MM level lead to calculated spectra that are in excellent agreement with experimental spectra (Figures 3–5). Clearly, treating H-bonding amino acids quantum mechanically will not lead to improved modeling of the experimental spectra. We have in fact undertaken calculations in which neutral UQ and the H-bonding amino acids are treated using QM, and we have found that the calculated spectra are very similar to that obtained when only neutral UQ is treated using QM (and the surrounding amino acids are treated using MM) (Zhao et al., J. Phys. Chem. B in press). Thus, it is very clear that, at least for the case of neutral quinones occupying the Q_A_ binding site, and as far as modeling isotope edited FTIR spectra is concerned, QM/MM calculations with only the quinone treated at the QM level need to be considered.

An experimental ^18^O isotope edited FTIR difference spectrum for UQ in the Q_A_ binding site is shown in Figure 5B. Three positive bands at 1660, 1629, and 1601 cm^−1^ are observed. By considering FTIR difference spectra obtained using PBRCs with unlabeled and specifically ^13^C_1_ and ^13^C_4_ labeled UQ occupying the Q_A_ binding site, it was concluded that the 1660 and 1601 cm^−1^ bands are due to the C_1_=O and C_4_=O vibrations of unlabeled neutral UQ, respectively. It was also concluded that the 1628 cm^−1^ band is due to a UQ C=C vibration (Breton et al., 1994c). Since the C_4_=O mode was so massively downshifted (from ~1660 cm^−1^ for UQ in solvent to 1601 cm^−1^ for UQ in the Q_A_ binding site) it was suggested that this group must be engaged in very strong hydrogen bonding, presumably with HisM219 (Figure 2) (Breton et al., 1994c). This conclusion is difficult to rationalize based on the crystal structural data and other experimental data [see Wraight and Gunner (2009) for a review]. Such a conclusion is also not supported by the data presented here. Specifically, the C_1_=O and C_4_=O modes of DMNQ and VK are found at 1653 and 1643 cm^−1^ (Table 2), respectively, compared to ~1662 cm^−1^ for the coupled C=O vibration in solution (Bandaranayake et al., 2006). Thus, the C_1_=O/C_4_= O mode of DMNQ or VK in the Q_A_ site is downshifted 9/19 cm^−1^, respectively, compared to that found in solution. Such shifts suggest that both C=O modes of DMNQ or VK are H-bonded in the Q_A_ site, albeit quite weakly.

From the experimental spectrum of VK (or DMNQ) in Figure 3B two positive bands are observed at 1651 and 1640 cm^−1^ and one negative band at 1620 cm^−1^. The two C=O modes of unlabeled VK give rise to the positive bands at 1651 and 1640 cm^−1^, but only a single band is observed at 1620 cm^−1^ upon ^18^O labeling. Two interpretations for these observations have been proposed (Breton et al., 1994b). One suggestion is that upon ^18^O labeling the 1640 cm^−1^ band downshifts to ~1620 cm^−1^, while the 1651 cm^−1^ band downshifts to near 1640 cm^−1^ and decreases in intensity. The negative band near 1640 cm^−1^ due to a C = ^18^O group of VK is then masked by the positive band (also at 1640 cm^−1^) due to the unlabeled C=O group. A second hypothesis is that the two C=O modes of unlabeled VK (at 1651 and 1640 cm^−1^) both downshift to ~1620 cm^−1^ upon ^18^O labeling. The different ^18^O induced shifts of the two C=O modes results from differential coupling to C=C modes.

The calculated data presented here allow us to address which of these interpretations could be appropriate, or if either is appropriate. The ONIOM calculations show that the C_1_=O and C_4_=O modes of unlabeled VK occur at 1652 and 1642 cm^−1^, and that neither of these modes are coupled to C=C modes (Table 2). Upon ^18^O labeling the C_1_=O/C_4_=O mode downshifts 24/18 cm^−1^, respectively. The modes of ^18^O labeled VK also display considerable coupling with C=C modes. Upon ^18^O labeling the C_1_=O/C_4_=O group couples with C=C_ring_ in-phase/out-of-phase vibrations, respectively. Coupling of the C_4_=O group to the out of phase C=C vibration leads to a large intensity enhancement, while coupling of the C_1_=O group to the in-phase C=C vibration leads to a large intensity decrease (Table 2). So, the calculations indicate that two separate uncoupled C=O modes in unlabeled VK give rise to predominantly a single mixed mode (that carries most of the intensity) in ^18^O labeled VK. These calculated results indicate that neither of the two previously proposed interpretations of the experimental spectra is correct. Clearly, the calculations presented here allow a more detailed insight into the nature of the bands in the experimental isotope edited FTIR difference spectra.

Experimentally, the vibrational modes of DMNQ are at a slightly higher frequency (~3 cm^−1^) than corresponding modes of VK. Presumably replacing the C_6_ methyl group with a phytyl unit causes this difference. Interestingly, this small frequency difference in the modes of DMNQ and VK is also found in our ONIOM calculated spectra (compare spectra in Figures 5C,B). This result is not entirely specific to the ONIOM method, however, as a small shift is also found in the gas phase calculations (Figure 3D).

The C_1_=O and C_4_=O modes of DMNQ and VK are calculated to be separated by 10 cm^−1^. This separation cannot be due to differences in the molecular group attached at C_6_. It must be due to differences in how the two C=O groups interact with the protein. Similarly, the two C=O modes of DQ are calculated to be separated by 13 cm^−1^, and this separation is also likely due to differences in how the two C=O groups of DQ interact with the protein.

For UQ_1_ (and MQ_0_) the separation of the C=O modes is ~32 cm^−1^ (1660–1628). Some of the differences in frequency of the C=O modes of UQ are due to the different orientation of the methoxy groups. If we assume that protein interactions with the C=O groups gives rise to a 13 cm^−1^ separation in the frequencies of the two C=O modes then this would indicate that the difference in the orientation of the methoxy groups of UQ (or MQ_0_) gives rise to a frequency difference of 19 cm^−1^ for the two C=O groups. This result is in quite good agreement with results from experimental spectra of UQ in solution, which show that the two C=O modes are separated by ~16 cm^−1^ (Breton et al., 1994c; Brudler et al., 1994).

Normal mode vibrational frequencies are governed by molecular bonding force constants. These force constants relate to the electronic structure of the molecule. Since the calculated and experimental spectra for DMNQ and VK are virtually the same it is concluded that the replacing the phytyl unit at C_6_ of VK with a methyl group does not appreciably perturb the electronic structure of the NQ ring. In addition, the tail does not perturb the protein in a way that significantly modifies any pigment protein interactions.

In VK and UQ_1_, the “kink” in the hydrocarbon chain after the first carbon atom is 3.1–3.2° higher in ONIOM calculations compared to gas phase calculations. It seems therefore, that the hydrocarbon chain is somewhat constrained relative to the quinone ring when incorporated into the Q_A_ binding site. It is not clear if this is a significant constraint, however. The C_1_=O and C_4_=O bonds of MQ_0_ and UQ_1_ (and of DMNQ and VK) are virtually unaltered in ONIOM calculations (Table 1). The hydrogen bond lengths for the C=O groups are also little altered (Table 1). The distance of the C_4_=O oxygen to the non heme iron atom is 0.029/0.055 Å longer for UQ_1_/VK compared to MQ_0_/DMNQ, respectively, suggesting a very small change in orientation of the UQ_1_/VK head-group (since the iron atom is fixed) compared to MQ_0_/DMNQ in the Q_A_ binding site. The hydrocarbon chain may therefore, lead to a very slight change in the orientation of the quinone ring in the Q_A_ binding site. Figure 2F shows that there is only a very small difference in the orientation of DMNQ relative to UQ_1_.

The experimental ^18^O isotope edited spectra for MQ_0_ and UQ_1_ are quite different (compare Figures 5B,C). This difference cannot be modeled computationally (Figure 5A). The experimental isotope edited spectrum for MQ_0_ is considerably noisier than the spectrum for UQ (Breton et al., 1994a). As far as we are aware the Q^−^_A_/Q_A_ FTIR difference spectrum for RCs with MQ_0_ in the binding site have never been reproduced, so the accuracy of the spectrum may be somewhat questionable. On the other hand MQ_0_ may be able to incorporate into the Q_A_ binding site with the methoxy groups oriented in several different ways. As described previously, each of these methoxy group conformers will have slightly different spectra (Lamichhane et al., 2010). This heterogeneity in orientation of MQ_0_ in the Q_A_ binding site may be a factor that contributes to differences in the experimental spectra for MQ_0_ and UQ, as demonstrated in Figures 5B,C. In spite of this, there are some overall similarities in the MQ_0_ and UQ_1_ experimental spectra in Figures 5B,C. For the MQ_0_ spectrum positive bands are found at 1665, 1631, and 1608 cm^−1^. For UQ_1_ the bands are at 1660, 1628, and 1601 cm^−1^. For the MQ_0_ spectrum negative bands are found at 1616 and 1602 cm^−1^. For UQ the bands are at 1613 and 1586 cm^−1^.

**Figure 6:**
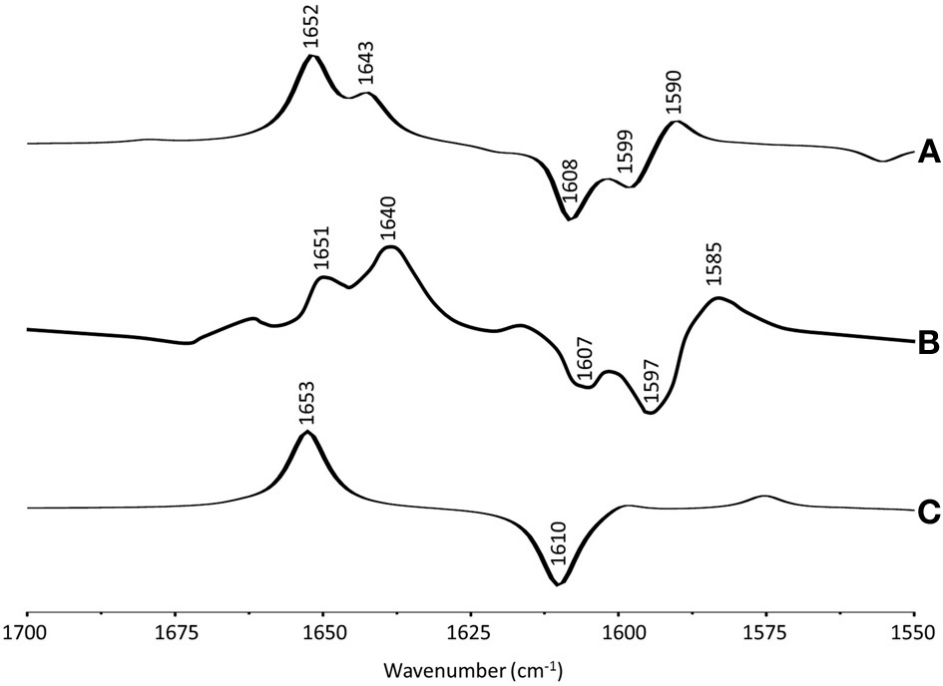
**(A)** ONIOM calculated ^13^C isotope edited DDS for neutral VK in the Q_A_ binding site. Experimental spectrum is also shown **(B)**, taken from (Breton, 1997) (with permission). **(C)** DFT calculated ^13^C isotope edited DDS for neutral VK in the gas phase. ONIOM and gas phase calculated spectra were scaled by 0.9718 and 0.9608, respectively.

The ONIOM calculated and experimental isotope edited spectra for VK are very similar, although the intensity ratios of the different bands do not appear to match well. For example, the intensity ratio of 1652 and 1644 cm^−1^ bands in the calculated spectrum (Figure 3A, dotted) is ~2.1, compared to a ratio of ~0.95 in the experimental spectrum. To investigate this further we have also calculated the ^13^C isotope edited spectrum for VK in the Q_A_ binding site (Figure 6A) (global ^13^C labeling of VK), and compared it to the corresponding experimental spectrum (Figure 6B). For completeness the calculated ^13^C isotope edited spectrum for VK in the gas phase is shown in Figure 6C. Again, it is evident that the calculated gas phase spectrum in no way resembles the experimental spectrum (compare Figures 6B,C). The normal modes (frequencies, intensities, and PEDs) that give rise to the various bands in the ONIOM calculated ^13^C isotope edited spectra of VK in Figure 6A are listed in Table 3.

**Table 3:** **Normal mode frequencies (in cm^−1^), intensities (in km/mol) and PEDs (in %) calculated using ONIOM methods for unlabeled and ^13^C labeled neutral VK**.

**Unlabeled**				**^13^C labeled**			
	**ν**	***I***	**Potential energy distribution**	**ν**	***I***	***Δν***	**Potential energy distribution**
VK	1652	224	C_1_=O (69)	1609	227	43	C1=O (73)
	1642	107	C_4_=O (60)	1598	116	44	C4=O (70)
	1611	54	C_5_=C_6_ = (60), −C_4_=O (8)	1555	43	56	C_5_=C_6_ (57)
	1591	96	C=C_arom_ (54), −C_4_=O (5)	1537	67	54	C=C_arom_ (55)

Frequency shifts upon ^13^C labeling are also listed. Negative signs in the PEDs refer to the relative phase of vibration of the internal coordinates. Only internal coordinates that contribute at least 5% are shown. Mode frequencies were scaled by 0.9718.

Notice that the ONIOM calculation predicts that the two C=O modes remain completely separate (do not mix) upon ^13^C labeling (Table 3), which is unlike the behavior observed for the C=O modes upon ^18^O labeling (see above). Also notice that in the experimental isotope edited spectrum (Figure 6B) the 1640 cm^−1^ band is now considerably more intense than the 1651 cm^−1^ band, whereas in the calculated spectrum the higher frequency band is considerably more intense. The origin of these mode intensity differences are not entirely clear and are currently being investigated by considering calculations associated with VK in the presence of various types of H-bonding molecules.

## Conclusions

We have shown that ONIOM type QM/MM calculations can be used to model experimental isotope edited FTIR difference spectra obtained using PBRCs that have had several different quinones incorporated into the Q_A_ binding site. The fact that the many different spectra can all be modeled is a clear indicator of the appropriateness of the approach.

The calculated spectra appear not to depend on whether the quinone incorporated has a prenyl/phytyl unit or a methyl group attached at C_6_. The electronic structure of the quinone ring is therefore, not sensitive to the presence or absence of a hydrocarbon side chain at C_6_. This suggests that the hydrocarbon side chain does not significantly constrain the quinone ring in the Q_A_ binding site.

Comparison of the calculated and experimental spectra, in combination with a consideration of the calculated PEDs of the normal modes, allows a direct assessment of the appropriateness of previous suggestions as to the origin of the bands in the experimental spectra.

### Conflict of interest statement

The authors declare that the research was conducted in the absence of any commercial or financial relationships that could be construed as a potential conflict of interest.

## Abbreviations

Ala: alanine
DS: difference spectra/spectrum spectroscopy or spectroscopic
C=O: carbonyl
DQ: duroquinone or 2,3,5,6-tetramethyl-1,4-benzoquinone
DFT: density functional theory
DMNQ: dimethyl naphthoquinone
ET: electron transfer
FTIR: Fourier Transform Infra-red
H-bond: hydrogen bond
His: histidine
MQ_0_: 2,3-dimethoxy-5,6-dimethyl-1,4-benzoquinone
MM: molecular mechanics
NQ: naphthoquinone
PQ: plastoquinone
PBRC: purple bacterial reaction center
PED: potential energy distribution
*Rb.*: *Rhodobacter*
QM: quantum mechanics
UQ_*n*_: ubiquinone-n
VK: vitamin K_1_ (phylloquinone).

## References

[B1] Bandaranayake K. Sivakumar V. Wang R. Hastings G. (2006). Modeling the A_1_ binding site in photosystem I. Density functional theory for the calculation of “Anion – Neutral” FTIR difference spectra of phylloquinone. Vib. Spectrosc. 42, 78–87 10.1016/j.vibspec.2006.01.003

[B2] Breton J. (1997). Efficient exchange of the primary quinone acceptor Q_A_ in isolated reaction centers of Rhodopseudomonas viridis. Proc. Natl. Acad. Sci. U.S.A. 94, 11318–11323 10.1073/pnas.94.21.11318 11038584 PMC23455

[B3] Breton J. Burie J. R. Boullais C. Berger G. Nabedryk E. (1994a). Binding sites of quinones in photosynthetic bacterial reaction centers investigated by light-induced FTIR difference spectroscopy: binding of chainless symmetrical quinones to the Q_A_ site of Rhodobacter sphaeroides. Biochemistry 33, 12405–12415 10.1021/bi00207a007 7918463

[B4] Breton J. Burie J. R. Berthomieu C. Berger G. Nabedryk E. (1994b). The binding sites of quinones in photosynthetic bacterial reaction centers investigated by light-induced FTIR difference spectroscopy: assignment of the Q_A_ vibrations in Rhodobacter sphaeroides using ^18^O- or ^13^C-labeled ubiquinone and vitamin K_1_. Biochemistry 33, 4953–4965 10.1021/bi00182a026 8161557

[B5] Breton J. Boullais C. Burie J. R. Nabedryk E. Mioskowski C. (1994c). Binding sites of quinones in photosynthetic bacterial reaction centers investigated by light-induced FTIR difference spectroscopy: assignment of the interactions of each carbonyl of Q_A_ in Rhodobacter sphaeroides using site-specific ^13^C-labeled ubiquinone. Biochemistry 33, 14378–14386 10.1021/bi00252a002 7981197

[B6] Breton J. Nabedryk E. (1996). Protein-quinone interactions in the bacterial photosynthetic reaction center: light-induced FTIR difference spectroscopy of the quinone vibrations. Biochim. Biophys. Acta. 1275, 84–90 10.1016/0005-2728(96)00054-0

[B7] Brudler R. de Groot H. J. van Liemt W. B. Steggerda W. F. Esmeijer R. Gast P. (1994). Asymmetric binding of the 1- and 4-C=O groups of QA in Rhodobacter sphaeroides R26 reaction centres monitored by Fourier transform infra-red spectroscopy using site-specific isotopically labelled ubiquinone-10. EMBO J. 13, 5523–5530 7988549 10.1002/j.1460-2075.1994.tb06889.x PMC395514

[B8] Case D. A. Cheatham T. E. Darden T. Gohlke H. Luo R. Merz K. M. (2005). The Amber biomolecular simulation programs. J. Comput. Chem. 26, 1668–1688 10.1002/jcc.20290 16200636 PMC1989667

[B9] Frisch M. J. Trucks G. W. Schlegel H. B. Scuseria G. E. Robb M. A. Cheeseman J. R. (2004). Gaussian 03, Revision C.02. Gaussian 03, Revision D.01.

[B10] Hale M. B. Blankenship R. E. Fuller R. C. (1983). Menaquinone is the sole quinone in the facultatively aerobic green photosynthetic bacterium *Chloroflexus aurantiacus*. Biochim. Biophys. Acta 723, 376–382 10.1016/0005-2728(83)90044-0

[B11] Hucke O. Schmid R. Labahn A. (2002). Exploring the primary electron acceptor (QA)-site of the bacterial reaction center from Rhodobacter sphaeroides. Binding mode of vitamin K derivatives. Eur. J. Biochem. 269, 1096–1108 10.1046/j.0014-2956.2001.02699.x 11856340

[B12] Ke B. (2001a). The bacterial photosynthetic reaction center: chemical composition and crystal structure, in Photosynthesis: Photobiochemistry and Photobiophysics, (Dordrecht; Boston: Kluwer Academic Publishers), 47–62

[B13] Ke B. (2001b). The stable primary acceptor QA and the secondary electron acceptor QB, in Photosynthesis: Photobiochemistry and Photobiophysics, (Dordrecht; Boston: Kluwer Academic Publishers), 289–304

[B14] Ke B. (2001c). The “Stable” primary electron acceptor (QA) of photosynthetic bacteria, in Photosynthesis: Photobiochemistry and Photobiophysics, (Dordrecht; Boston: Kluwer Academic Publishers), 101–110

[B15] Ke B. B. (2001d). The secondary electron acceptor (QB) of photosynthetic bacteria, in Photobiochemistry and Photobiophysics, (Dordrecht; Boston: Kluwer Academic Publishers), 111–128

[B16] Lamichhane H. P. Hastings G. (2011). Calculated vibrational properties of pigments in protein binding sites. Proc. Natl. Acad. Sci. U.S.A. 108, 10526–10531 10.1073/pnas.1104046108 21670247 PMC3127941

[B17] Lamichhane H. P. Hastings G. (2013). Calculated vibrational properties of ubisemiquinones. Comput. Biol. J. 2013:11 10.1155/2013/807592

[B18] Lamichhane H. Wang R. Hastings G. (2010). Comparison of calculated and experimental FTIR spectra of specifically labeled ubiquinones. Vib. Spectrosc. 55, 279–286 10.1016/j.vibspec.2010.12.008

[B19] Lamichhane H. Wang R. L. Hastings G. (2011). Comparison of calculated and experimental FTIR spectra of specifically labeled ubiquinones. Vib. Spectrosc. 55, 279–286 10.1016/j.vibspec.2010.12.008

[B20] Lin T. O'Malley P. (2008). An ONIOM study of the Q(A) site semiquinone in the Rhodobacter sphaeroides photosynthetic reaction centre. J. Mol. Struct. Theochem. 870, 31–35 10.1016/j.theochem.2008.08.034

[B21] Martin J. M. L. Van Alsenoy C. (1995). GAR2PED. Antwerp: University of Antwerp

[B22] McComb J. C. Stein R. R. Wraight C. A. (1990). Investigations on the influence of headgroup substitution and isoprene side-chain length in the function of primary and secondary quinones of bacterial reaction centers. Biochim. Biophys. Acta 1015, 156–171 10.1016/0005-2728(90)90227-U 2404516

[B23] Noguchi T. Berthomieu C. (2005). Molecular analysis by vibrational spectroscopy, in Photosystem II The Light Driven Water:Plastoquinone Oxidoreductase, eds Wydrzynski T. Satoh K. (Dordrecht: Springer), 367–387

[B24] Parameswaran S. Wang R. Hastings G. (2008). Calculation of the vibrational properties of chlorophyll a in solution. J. Phys. Chem. B 112, 14056–14062 10.1021/jp806115q 18842020

[B25] Shopes R. J. Wraight C. A. (1985). The acceptor quinone complex of Rhodopseudomonas viridis reaction centers. Biochim. Biophys. Acta 806, 348–356 10.1016/0005-2728(85)90242-7 2982395

[B26] Sinnecker S. Flores M. Lubitz W. (2006). Protein-cofactor interactions in bacterial reaction centers from Rhodobacter sphaeroides R-26: effect of hydrogen bonding on the electronic and geometric structure of the primary quinone. A density functional theory study. Phys. Chem. Chem. Phys. 8, 5659–5670 10.1039/b612568a 17149487

[B27] Srinivasan N. Golbeck J. H. (2009). Protein-cofactor interactions in bioenergetic complexes: the role of the A1A and A1B phylloquinones in Photosystem I. Biochim. Biophys. Acta 1787, 1057–1088 10.1016/j.bbabio.2009.04.010 19409369

[B28] Stowell M. H. McPhillips T. M. Rees D. C. Soltis S. M. Abresch E. Feher G. (1997). Light-induced structural changes in photosynthetic reaction center: implications for mechanism of electron-proton transfer. Science 276, 812–816 10.1126/science.276.5313.812 9115209

[B29] Trumpower B. (1982). Function of Quinones in Energy Conserving Systems. New York, NY: Academic Press

[B30] Vreven T. Byun K. S. Komaromi I. Dapprich S. Montgomery J. A. Morokuma K. (2006). Combining quantum mechanics methods with molecular mechanics methods in ONIOM. J. Chem. Theory Comput. 2, 815–826 10.1021/ct050289g 26626688

[B31] Warncke K. Gunner M. R. Braun B. S. Gu L. Q. Yu C. A. Bruce J. M. (1994). Influence of hydrocarbon tail structure on quinone binding and electron-transfer performance at the Q(a) and Q(B) sites of the photosynthetic reaction-center protein. Biochemistry 33, 7830–7841 10.1021/bi00191a010 8011647

[B32] Wheeler R. A. (2001). Quinones and quinoidal radicals in photosynthesis, in Theoretical Biochemistry-Processes and Properties of Biological Systems, ed Eriksson L. A. (Amsterdam: Elsevier), 655–690 10.1016/S1380-7323(01)80016-9

[B33] Wraight C. A. Gunner M. R. (2009). The acceptor quinones of purple photosynthetic bacteria—structure and spectroscopy, in The Purple Photosynthetic Bacteria, eds Hunter C. Daldal F. Thurnauer M. Beatty T. (Dordrecht: Springer), 379–405

